# P-1909. Education Quality of YouTube Videos on MRSA

**DOI:** 10.1093/ofid/ofaf695.2078

**Published:** 2026-01-11

**Authors:** Nicholas F Angelino, Christopher Damiani, Madeleine Purcell, Chad Sussman, Max Jacobs, Shatha AlShanqeeti, Devang M Patel

**Affiliations:** University of Maryland School of Medicine, Baltimore, MD; University of Maryland School of Medicine, Baltimore, MD; University of Maryland School of Medicine, Baltimore, MD; University of Maryland School of Medicine, Baltimore, MD; University of Maryland School of Medicine, Baltimore, MD; University of Maryland, Baltimore, MD; University of Maryland School of Medicine, Baltimore, MD

## Abstract

**Background:**

With the growing availability of online health resources, patients can turn to the internet for information about medical conditions. However, the accuracy, reliability, and quality of this content vary widely. YouTube hosts numerous videos on methicillin-resistant *Staphylococcus aureus* (MRSA), produced by healthcare professionals, patients, and media sources. This study assessed the quality and reliability of MRSA-related YouTube videos, as rated by medical students, residents, infectious disease fellows, and an infectious disease attending.

**Figure 1:**
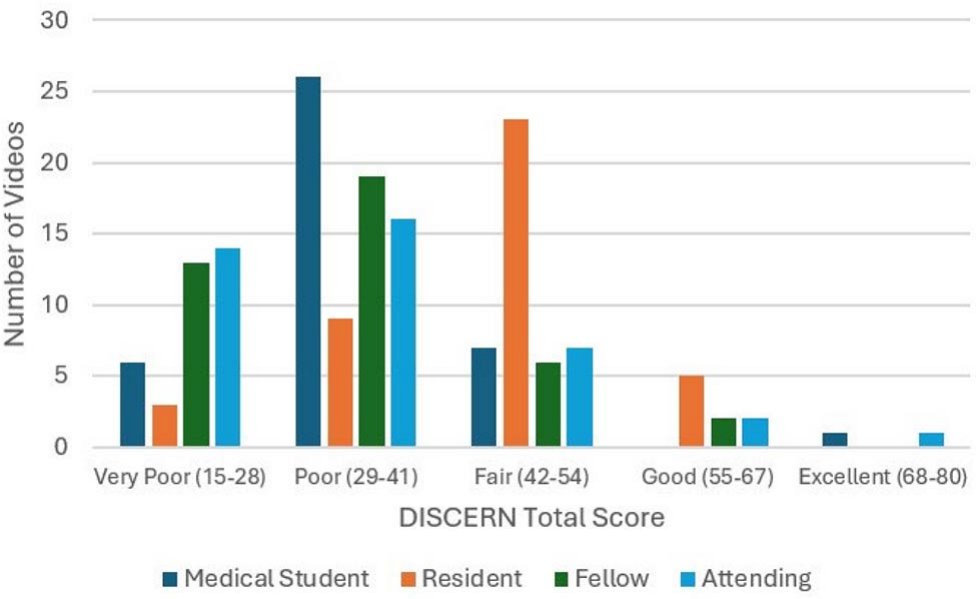
Mean total DISCERN score of MRSA YouTube videos rated by four levels of medical training. Mean total scores of medical students, residents, and fellows were recorded as the average of scores within the group.

**Table 1:**
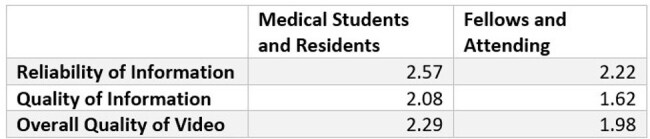
Mean question level DISCERN score of three topic categories of lower-level training groups compared to higher-level training groups. Reliability of information was covered by Q1-8 of DISCERN, quality of information was covered by Q9-15 of DISCERN, and overall quality of the video was covered by Q16 of DISCERN. Each question was scored 1-5.

**Methods:**

A YouTube search for "MRSA" identified the top 40 videos based on the “relevance” search qualifier. Two medical students, two internal medicine residents, two infectious disease fellows, and one infectious disease attending independently reviewed each video using DISCERN, a validated tool for evaluating online health information. The tool includes 16 questions rated on a 5-point scale. The questions can be separated into topic categories of reliability of information (Q1-8), quality of information (Q9-15), and overall quality of the video (Q16). Total DISCERN scores categorize videos as 15–28 (very poor), 29–41 (poor), 42–54 (moderate), 55–67 (good), and 68–80 (excellent).

**Results:**

The videos were predominantly rated as low-quality (Figure 1). The mean total DISCERN score across all videos was 34.4 out of 80, which is “poor.” Table 1 shows the mean question level score of the different topic categories. The table compares the two lower-level training groups, medical students and residents, to the two higher-level training groups, fellows and the attending. The higher-level training groups were associated with more critical reviews of the videos.

**Conclusion:**

MRSA-related YouTube videos are widely available, but generally low in educational quality. Healthcare professionals and patients should approach online medical content with caution. Improved standards and oversight for digital health information are essential to ensure patients receive accurate, reliable, and high-quality educational resources.

**Disclosures:**

All Authors: No reported disclosures

